# Efficacy evaluation of 2D, 3D U-Net semantic segmentation and atlas-based segmentation of normal lungs excluding the trachea and main bronchi

**DOI:** 10.1093/jrr/rrz086

**Published:** 2020-02-11

**Authors:** Takafumi Nemoto, Natsumi Futakami, Masamichi Yagi, Atsuhiro Kumabe, Atsuya Takeda, Etsuo Kunieda, Naoyuki Shigematsu

**Affiliations:** 1 Division of Radiation Oncology, Saiseikai Yokohamashi Tobu-Hospital, Shimosueyoshi 3-6-1, Tsurumi-ku, Yokohama-shi, Kanagawa, 230-8765, Japan; 2 Department of Radiation Oncology, Tokai University School of Medicine, Shimokasuya 143, Isehara-shi, Kanagawa, 259-1143, Japan; 3 Department of Radiology, Keio University School of Medicine, Shinanomachi 35, Shinjyuku-ku, Tokyo, 160-8582, Japan; 4 HPC&AI Business Dept., Platform Technical Engineer Div., System Platform Solution Unit, Fujitsu Limited, World Trade Center Building, 4-1, Hamamatsucho 2-chome, Minato-ku, Tokyo, 105-6125, Japan; 5 Radiation Oncology Center, Ofuna Chuo Hospital, Kamakura, 247-0056, Japan

**Keywords:** semantic segmentation, U-Net, lung cancer, trachea, main bronchi

## Abstract

This study aimed to examine the efficacy of semantic segmentation implemented by deep learning and to confirm whether this method is more effective than a commercially dominant auto-segmentation tool with regards to delineating normal lung excluding the trachea and main bronchi. A total of 232 non-small-cell lung cancer cases were examined**.** The computed tomography (CT) images of these cases were converted from Digital Imaging and Communications in Medicine (DICOM) Radiation Therapy (RT) formats to arrays of 32 × 128 × 128 voxels and input into both 2D and 3D U-Net, which are deep learning networks for semantic segmentation. The number of training, validation and test sets were 160, 40 and 32, respectively. Dice similarity coefficients (DSCs) of the test set were evaluated employing Smart Segmentation^Ⓡ^ Knowledge Based Contouring (Smart segmentation is an atlas-based segmentation tool), as well as the 2D and 3D U-Net. The mean DSCs of the test set were 0.964 [95% confidence interval (CI), 0.960–0.968], 0.990 (95% CI, 0.989–0.992) and 0.990 (95% CI, 0.989–0.991) with Smart segmentation, 2D and 3D U-Net, respectively. Compared with Smart segmentation, both U-Nets presented significantly higher DSCs by the Wilcoxon signed-rank test (*P* < 0.01). There was no difference in mean DSC between the 2D and 3D U-Net systems. The newly-devised 2D and 3D U-Net approaches were found to be more effective than a commercial auto-segmentation tool. Even the relatively shallow 2D U-Net which does not require high-performance computational resources was effective enough for the lung segmentation. Semantic segmentation using deep learning was useful in radiation treatment planning for lung cancers.

## INTRODUCTION 

Lung cancer is the most common malignancy in both men and women. In 2018, 1.8 million people died worldwide as a result of this disease, which is the most frequent cause of cancer-related deaths globally as well as in Japan [1, 2]. According to the National Comprehensive Cancer Network (NCCN) guidelines, chemoradiation therapy is recommended for unresectable stage II and III non-small cell lung cancer (NSCLC), and stereotactic body radiotherapy for unresectable early stage NSCLC [3]. In addition, consolidation therapy with the anti-programmed death ligand 1 antibody durvalumab after concurrent chemoradiation therapy in unresectable stage III NSCLC was reported to significantly extend progression-free survival as compared to a placebo [4]. Therefore, an increase in NSCLC patients undergoing radiation therapy is anticipated. Radiation pneumonitis is a common complication of radiation therapy in NSCLC patients. Thus, exposure of the normal lung needs to be limited. In efforts to reduce the risk of radiation pneumonitis, V20 (the lung volume receiving ≥20 Gy) ≤30–35% and mean lung dose (MLD) ≤20–23 Gy have been recommended [5].

In light of the above considerations, it is essential to determine the extent of normal lung exposure in radiation treatment planning for lung cancer patients. Therefore, we need to obtain accurate contour delineations of the tumor and organs at risk (OARs) on computed tomography (CT) images for radiotherapy planning. If the delineations are incorrect, the dose calculation results would potentially be unreliable, because the calculations are based on these delineations. Manual delineations by radiation oncologists are generally time-consuming [6], and inter- and intra-observer segmentation variability occurs [7], highlighting the need for improvement in delineation techniques. The auto-segmentation tool will reduce the time needed to achieve accurate delineations and eliminate inter- and intra-observer segmentation variability [8, 9]. Commercial tools with atlas-based segmentation or model-based segmentation are currently available [10]. However, these tools are not fully automated and do not consistently provide the intended delineations. For example, according to the RTOG 1106 guideline, the trachea and main bronchi are necessarily excluded from normal lung delineation when planning lung cancer radiation treatment [11]. However, a widely-available commercial auto-segmentation tool, the Smart Segmentation^Ⓡ^ Knowledge Based Contouring (Varian Medical Systems, Palo-Alto, CA, USA; hereinafter referred to as Smart segmentation) system, includes the trachea or main bronchi in normal lung delineation. Therefore, the obtained results must subsequently be corrected manually. For this reason, there is an urgent need for alternative auto-segmentation techniques producing more accurate delineation of the normal lung.

The application of artificial intelligence, such as auto-segmentation techniques, has been drawing increasing attention for both clinical applications and research. Convolutional neural networks (CNN), especially, have reportedly been applied to contour delineations of OARs [12], potentially solving many of the problems encountered in achieving optimal delineation. CNN recognizes objects from medical images by combining the convolutional and pooling layers, such that semantic segmentations can be implemented. Among the available CNN, U-Net [13], which has a U-shaped encoder–decoder structure, down-samples and up-samples the original images, thereby extracting a feature map of the same size as the original image, and can thus segment images. Since U-Net is characterized by integrating both local features and general location information of the object, it is expected to be effective in the delineations required for radiation treatment planning. It can be implemented in either a 2D or a 3D format, and each has both advantages and disadvantages. With 2D U-Net, the 3D direction of information is decreased since each image is handled independently. However, this system can learn a large number of samples. With 3D U-Net, the number of samples is smaller, which means that the amount of information per sample is increased, while the 3D direction of information is enriched.

Several studies have focused on semantic segmentation of lung tissues on CT images using 2D or 3D U-Net [14–17]. However, to our knowledge, there are no reports on the differences between U-Net and existing auto-segmentation tools using the same dataset. Furthermore, the 2D and 3D U-Net approaches, applied under similar conditions using the same dataset, have not been compared.

We therefore attempted semantic segmentation of lung CT images using both 2D and 3D U-Net, then examined their efficacies in comparison with that of the existing Smart segmentation. We also compared the utilities of the 2D and 3D U-Net with each other.

## MATERIALS AND METHODS 

### Datasets

The Cancer Imaging Archive (TCIA) is an open access database of medical images for cancer research. We examined 232 NSCLC cases, from LUNG 1–001 to LUNG 1–232 comprising the NSCLC-Radiomics [18], a CT dataset of NSCLC cases published by the TCIA. This dataset consists of 3 mm thick slices and includes the entire chest. Of the total 232 cases, 200 were assigned to a training and validation set, while the remaining 32 cases were randomly chosen as a test set to evaluate the accuracy of predicting results for unknown data.

The Radiation Therapy Oncology Group (RTOG) 1106 contouring atlas guideline recommends that gross tumor volume, the hilar portions of the lungs and the trachea/main bronchi not be included in the lung. A ground truth value for the lungs is obtained by contouring, according to the RTOG1106, as the whole lung excluding the trachea and main bronchi, although exclusion of secondary bronchi and small vessels near the hila cannot be guaranteed. In all 232 cases the ground truth values were determined manually by one radiologist and one medical physician using Eclipse version 13.6 (Varian Medical Systems, Palo-Alto, CA, USA). In addition, 32 cases were contoured employing Smart segmentation version 13.6 summarizing the contour structures of the right and left lungs into one whole-lung structure as the control group for the test set, allowing comparison between U-Net and an established auto-segmentation tool, Smart segmentation.

**Fig. 1.**
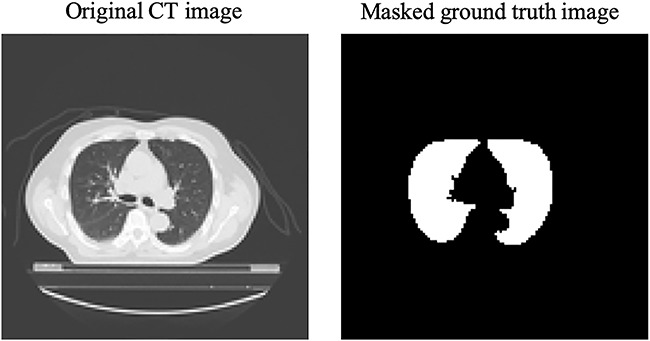
The left image is an original CT scan and the right image is a masked ground truth image showing the lungs as white and other areas as black.

The CT images and contour data were exported as Digital Imaging and Communications in Medicine (DICOM) data and converted to PNG images. Masked images in which the lungs are white and the non-lung areas are black were created from the contour data. Sample images of an original CT and a ground truth image are presented in Fig. 1. All images were resized from 512 × 512 to 128 × 128 pixels and each case was fixed at 32 images.

Cross-validation is a model validation technique used to evaluate machine learning models in order to make such models more generalizable. A 5-fold cross-validation was performed, in which 200 cases were randomly partitioned into five equal size subsamples, such that 40 cases and learnings were repeated five times, with interchange of the 40 cases assigned as the validation set and the 160 cases assigned as the training set.

### U-Net models

**Fig. 2.**
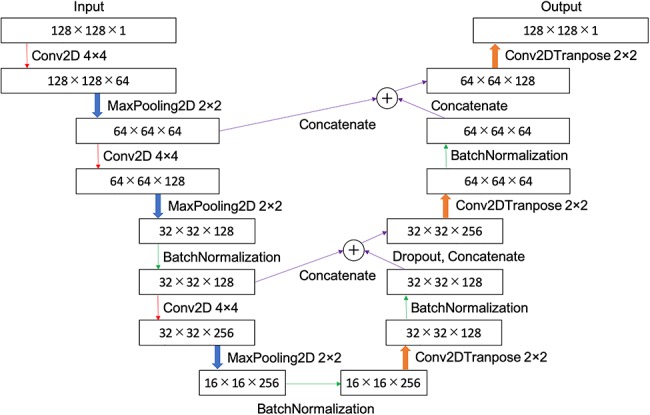
Schemaic diagram of 2D U-Net: a CT image of 128 × 128 pixels was input, down-sampled to 16 × 16, and then up-sampled.

**Fig. 3.**
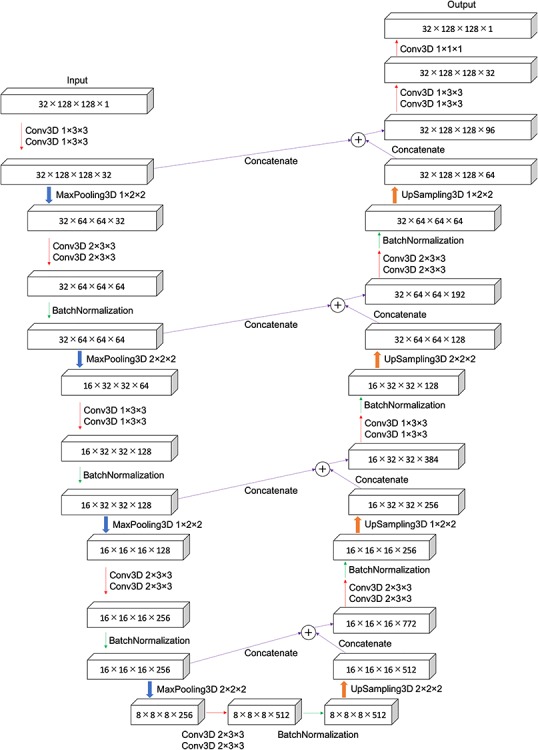
Schemtic diagram of 3D U-Net: a 3D CT image of 32 × 128 × 128 voxels was input, down-sampled to 8 × 8 × 8, and then up-sampled.

The U-Net models devised are presented in Figs 2 and 3. The words in these figures describe the methods of Keras. Fig. 2 shows the 2D U-Net model. The input layer contained 128 × 128 pixels with 1 channel. We performed 2D convolution (Conv2D) by applying a 2 × 2 filter to the input, in which zero padding filled the perimeter of the input with 0 compensating for the size reduction produced by the filter. Max pooling employing MaxPooling2D, by which the maximum value is selected from each region and then compressed, was performed 2-dimensionally to reduce pixel size. Conv2DTranspose is an operation which is the opposite of that used for Conv2D, whereby pixel size is increased using a 2 × 2 pixels filter. Batch Normalization (BatchNormalization) is a process by which biased output distribution, obtained from the previous layer, is corrected. Dropout is a process by which a portion of the units are randomly deactivated. Random deactivation was achieved in 50% of the units in this study. Concatenation is a process applied in order to connect the input arrays.

Fig. 3 shows the 3D U-Net model. The input layer contained 32 × 128 × 128 voxels with 1 channel. We performed 3D convolution (Conv3D) by applying a 1 × 3 × 3, 2 × 3 × 3, 1 × 1 × 1 filter to the input with the appropriate zero padding, the same conditions as those applied for the 2D contouring. MaxPooling3D is a max pooling technique performed 3-dimensionally. The 3D up-sampling (UpSampling3D) doubled the voxel size along all dimensions.

For both 2D and 3D U-Nets, rectified linear unit (ReLU) was used for the input and hidden layers, and sigmoid function for the output layer as the activation function. Data augmentation is a method for increasing the number of data points, thereby contributing to both the prevention of over-fitting and enhanced performance. In this study, a left-to-right inversion and a white-to-black inversion were produced to achieve data augmentation producing a 4-fold increase in the original data volume. An adaptive moment estimation (Adam) was applied as an optimizer. The optimum rate of learning was selected by changing the learning rates along a broad range of values, including 0.3, 0.1, 0.03, 0.01, 0.003, 0.001, 0.003 and 0.001. A loss function is calculated as the difference between the ground truth and the predicted output, which requires to be minimized for optimization. It was calculated employing the following equations:

(1) \begin{equation*} \mathrm{Loss}\ \mathrm{function}=2-\left\{ IOU\left(A,B\right)+ DSC\left(A,B\right)\right\} \end{equation*}

(2) \begin{equation*} IOU\left(A,B\right)=\frac{\left|A\cap B\right|}{\left|A\cup B\right|} \end{equation*}

(3) \begin{equation*} DSC\left(A,B\right)=\frac{2\left|A\cap B\right|}{\left|A\right|+\left|B\right|} \end{equation*}

where, *A* is a true value and *B* is an estimated value. The Jaccard coefficient is expressed as intersection over union (IoU) and the dice similarity coefficient as DSC. The Jaccard coefficient and the Dice coefficient are both indicators for assessing the degree of similarity between classes.

The accuracy of contour delineation was assessed using DSC. The DSC for U-Net was obtained based on the ensemble learning for five inference results obtained from a 5-fold cross validation. Ensemble learning is illustrated by the following example: when a CT pixel value is 1 for three inferences and 0 for two inferences, 1 is chosen as the value representing the majority.

Calculation processes were implemented on the operating system consisting of the following components: Windows10 Pro, CPU: Intel^Ⓡ^ Xeon^Ⓡ^ CPU E5–2667 v3 (Intel Corp., CA, USA), memory: 64.0 GB, graphics processing unit (GPU): Quadro M5000 8.0 GB 1 piece + Quadro P5000 16.0 GB 1 piece (NVIDIA Corp., CA, USA). Masked images were created by Python 3.5.6, NumPy 1.6.1, OpenCV 3.1.0 and Pillow 5.2.0. U-Nets were constructed employing Python 3.5.6, NumPy 1.6.1 and Keras 2.2.4 with TensorFlow-GPU 1.12.0 as the backend on Compute Unified Device Architecture (CUDA) 9.0.176 and the NVIDIA CUDA^Ⓡ^ Deep Neural Network library (cuDNN) 7.3.1.

To evaluate the dice similarity coefficient (DSC) of Smart segmentation, and those of the 2D and 3D U-Net systems for the test set consisting of 32 cases, we applied the Wilcoxon signed-rank test. A *P*-value <0.05 was considered to indicate a statistically significant difference. All statistical analyses were performed with EZR 1.40 [19] (Saitama Medical Center, Jichi Medical University, Saitama, Japan), which is a graphical user interface for R (The R Foundation for Statistical Computing, Vienna, Austria).

## RESULTS

The optimum learning rate was found to be 0.001 for 2D U-Net and 0.0001 for 3D U-Net. It takes ~13 h for every learning rate to learn 2D U-Net of 300 epochs, and ~11 h for every learning rate to learn 3D U-Net of 50 epochs.

Table 1 shows the mean DSC with the standard deviation, 95% confidence interval (CI), maximum, median and minimum of DSC for each technique. These values were calculated for the test set of 32 cases. Smart segmentation yielded a mean DSC of 0.964, while 2D and 3D U-Net both had a mean DSC of 0.990. Thus, the 2D and 3D U-Net had a mean DSC ~2.7% higher than that of Smart segmentation. Furthermore, Table 1 presents comparisons among the *P*-values for Smart segmentation, 2D and 3D U-Net. Both U-Nets differed significantly from Smart segmentation, at *P* < 0.01.

**Fig. 4.**
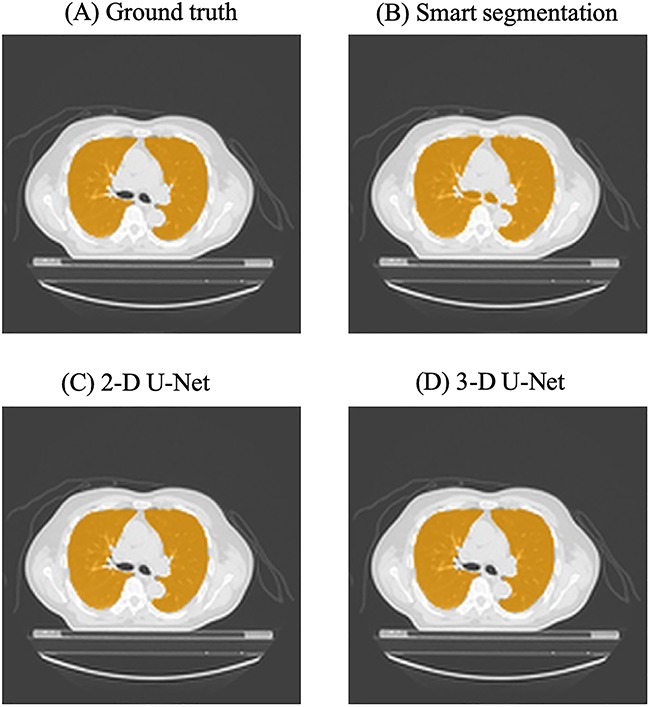
Lung regions are indicated in orange in the same CT images, obtained at the level of the main bronchi. (**A**) Ground truth image, drawn manually, (**B**) an image generated by Smart segmentation, (**C**) and (**D**) predictions based on 2D and 3D U-Net.

Fig. 4 shows the main bronchi present in a slice selected arbitrarily from the test set of 32 cases. Compared with the ground truth image, Smart segmentation depicted the main bronchi and other structures incorrectly, while 2D and 3D U-Net showed all but the main bronchi correctly.

## DISCUSSION

Minimizing normal lung dose represented with V20 or MLD is important for preventing radiation pneumonitis when treating NSCLC. To achieve accurate dose evaluation of normal pulmonary tissue, it is necessary to precisely delineate the normal lung parenchyma excluding the trachea and main bronchi. In this study, we devised U-Net models of the lung to assess their efficacy in performing contour delineations. We also endeavored to confirm whether this method is more effective than a widely-used atlas-based segmentation tool (Smart segmentation) for delineating the lung. The CT images of 232 NSCLC cases were input into both 2D and 3D U-Net systems, which are deep learning networks designed for semantic segmentation. Training, validation and test sets were employed to allow thorough assessment of the results. DSCs of the test set were obtained employing Smart segmentation and both the 2D and the 3D U-Net systems.

Both U-Nets yielded significantly higher DSCs than the commercially available semantic segmentation tool ( *P*< 0.01). We thus conclude that the newly-devised 2D and 3D U-Net systems are both more effective for delineating pulmonary structures than the commercial auto-segmentation tool. Semantic segmentation using deep learning has the potential to be very useful when planning radiation treatments for lung cancer patients. Improving the accuracy of lung delineation is anticipated to enhance delivery of radiation to malignant sites, while limiting exposure of the normal lung tissue to radiation and thereby reducing the incidence of radiation pneumonitis and other complications. The clinical potential, as the well as the likely cost savings, of these approaches merit further study.

Furthermore, there was no difference in mean DSC between the 2D and 3D U-Net systems, indicating similar accuracy in contour delineation. Although semantic segmentation using a deep learning 2D network lacks craniocaudal information, this apparent deficiency is speculated to have no effect on contour delineation of the lung. There are several human organs with longitudinally-oriented structures, like the lung. Therefore, our 2D U-Net system that allows the contour delineation is considered to provide useful practical knowledge.

Park *et al*. performed semantic segmentation with 3D U-Net in individual pulmonary lobes, reporting a DSC of 0.9680 ± 0.018 [16]. In their study, the convolution filter size of the 3D U-Net was 3 × 3 × 3, applied in a uniform manner, and the maximum number of channels was set at 576. On the other hand, in our present study, the filter sizes of the 3D U-Net were variable (i.e., 1 × 3 × 3, 2 × 3 × 3 and 1 × 1 × 1) and the maximum number of channels was 772. Characteristic maps to be visualized increase as the number of channels rises. Therefore, the 3D U-Net devised for this study might be able to yield high DSC. Furthermore, the DSC in this study was higher even with the 2D U-Net than that obtained from the 3D U-Net used by Park *et al*. In this study, the 2D U-Net system considered the information at the image margin, since zero padding had been applied for learning, and suppressed over-fitting due to drop-out, which in turn increased DSC.

3D U-Net requires enormous computational resources and a longer calculation time, as compared to 2D U-Net. When a high-performance computational resource is required, calculation on a cloud server is generally performed. However on-line processing via the public network for medical application may raise ethical or security concerns. Therefore, the calculation device should be installed on-premises within the hospital. Medical applications requiring immediate processing need a smaller calculation amount. Reducing computation quantity without lowering precision enables edge equipment and embedded systems in medicine. Therefore, this study is of value because 2D U-Net was found to yield results similar to those of 3D U-Net.

**Table 1:** Performance and comparison of each technique calculated for test set in DSC.

Technique	Mean DSC	95%CI of DSC	Maximum DSC	Median DSC	Minimum DSC	vs Smart segmention *P*-value
Smart segmention	0.964 ± 0.011	0.960–0.968	0.978	0.967	0.924	not applicable
2D U-Net	0.990 ± 0.004	0.989–0.992	0.994	0.991	0.976	<0.01
3D U-Net	0.990 ± 0.002	0.989–0.991	0.993	0.991	0.982	<0.01

We found that both 2D and 3D U-Nets allow accurate contour delineation of the lung excluding the main bronchi, while Smart segmentation does not delineate the main bronchi accurately. Dong *et al*. predicted lung morphology in accordance with RTOG 1106 for OARs, based on atlases, using the U-Net-generative adversarial network (U-Net-GAN) [17, 20]. U-Net-GAN incorporates U-Net into discriminators of GAN, such that DSC was 0.97 ± 0.01 for the left lung and 0.97 ± 0.01 for the right lung in their study [17]. Our U-Net, developed for this study, yielded higher DSC than that obtained by Dong *et al*. Based on the RTOG 1106 atlases, contour delineation of the lung excluding the trachea and main bronchi was achieved in this study. We performed data augmentation prior to processing by producing a left-to-right inversion or a white-to-black inversion. The efficacy of U-Net implementation was validated under various conditions including the layer structure, batch normalization and drop-out. In general, the U-Net-GAN used by Dong *et al*. allows complex learning and high accuracy can be anticipated. However, our present study revealed that U-Net alone, when applied appropriately, even without using GAN achieves accurate prediction. Furthermore, our 2D U-Net which down-sampled three times was shallower than the U-Net devised by Ronneberger *et al*. which down-sampled four times [13]. This means that even the relatively shallow 2D U-Net was effective enough for the lung segmentation.

It is reported that existing auto-segmentation tools reduce delineation time and eliminate inter- and intra-observer segmentation variability [8, 9]. U-Net segmentation tools will be more effective regarding these things in clinical practice, while we have to keep a more watchful eye on their performance and quality.

Another study addressed the reduction of radiation doses in highly functional lung regions by evaluating lung function with functional pulmonary imaging [21]. Future research focusing on the achievement of segmentation that predicts lung function would be extremely beneficial for preventing radiation-induced lung injury.

In conclusion, we validated the efficacy of semantic segmentation of the lung excluding the trachea and main bronchi using 2D and 3D Net systems. The results revealed that both U-Net systems yielded higher accuracy than the conventional method and had the same mean DSC (0.990). Our newly devised approach was useful for increasing the accuracy of lung contour delineation.

2D and 3D U-Nets did not differ in terms of the accuracy of contour delineation of the lung. Considering that several human organs have longitudinally-oriented structures, like the lung, even relatively shallow 2D delineation may provide useful information allowing the lung contour to be delineated with accuracy sufficient for clinical purposes. Thus, we believe that our novel 2D U-Net system has practical medical applications.
